# Development and Characterization of Anti-Nitr9 Antibodies

**DOI:** 10.1155/2012/596925

**Published:** 2012-09-24

**Authors:** Radhika N. Shah, Ivan Rodriguez-Nunez, Donna D. Eason, Robert N. Haire, Julien Y. Bertrand, Valērie Wittamer, David Traver, Shila K. Nordone, Gary W. Litman, Jeffrey A. Yoder

**Affiliations:** ^1^Department of Molecular Biomedical Sciences and Center for Comparative Medicine and Translational Research, College of Veterinary Medicine, North Carolina State University, 1060 William Moore Drive, Raleigh, NC 27607, USA; ^2^Immunology Program, College of Veterinary Medicine, North Carolina State University, 1060 William Moore Drive, Raleigh, NC 27607, USA; ^3^Children's Research Institute, Department of Pediatrics, University of South Florida College of Medicine, 140 Seventh Avenue South, St. Petersburg, FL 33701, USA; ^4^Immunology Program, H. Lee Moffitt Cancer Center and Research Institute, 12902 Magnolia Avenue, Tampa, FL 33612, USA; ^5^Department of Pathology and Immunology, University of Geneva School of Medicine, Rue Michel-Servet 1, 1211 Geneva 4, Switzerland; ^6^Department of Cellular and Molecular Medicine and Section of Cell and Developmental Biology, University of California at San Diego, 9500 Gilman Drive, La Jolla, CA 92093-0380, USA; ^7^Department of Molecular Genetics, All Children's Hospital, 501 Sixth Avenue South, St. Petersburg, FL 33701, USA

## Abstract

The novel immune-type receptors (NITRs), which have been described in numerous bony fish species, are encoded by multigene families of inhibitory and activating receptors and are predicted to be functional orthologs to the mammalian natural killer cell receptors (NKRs). Within the zebrafish NITR family, *nitr9* is the only gene predicted to encode an activating receptor. However, alternative RNA splicing generates three distinct *nitr9* transcripts, each of which encodes a different isoform. Although *nitr9* transcripts have been detected in zebrafish lymphocytes, the specific hematopoietic lineage(s) that expresses Nitr9 remains to be determined. In an effort to better understand the role of NITRs in zebrafish immunity, anti-Nitr9 monoclonal antibodies were generated and evaluated for the ability to recognize the three Nitr9 isoforms. The application of these antibodies to flow cytometry should prove to be useful for identifying the specific lymphocyte lineages that express Nitr9 and may permit the isolation of Nitr9-expressing cells that can be directly assessed for cytotoxic (e.g., NK) function.

## 1. Introduction

Mammalian natural killer (NK) cells are large, granular lymphocytes of the innate immune system that express several cell surface receptors to regulate cytotoxic function through a complex network of signaling pathways. NK cell receptors include both activating and inhibitory forms that are proficient in distinguishing neoplastic or virally infected cells from normal host cells [1, 2]. The regulation of NK cell cytotoxicity is dependent on the integration of signals from activating and inhibitory receptors [3]. Although it is postulated that NK cell receptors arose early in vertebrate phylogeny, functional data are based primarily on studies of mammalian NK cell receptors [4].

In order to appreciate the origins and evolution of NK cell receptors and their function, it is critical to define equivalent receptor forms in nonmammalian species. The bony fish represent one of the earliest vertebrate lineages with a functional innate and adaptive immune response that closely parallels that of humans and other mammals [5]. A large multigene family of recently and rapidly evolving inhibitory and activating novel immune-type receptors (NITRs) that share structural and functional characteristics with mammalian NK cell receptors has been identified in multiple fish species [6, 7]. Complete analyses of the NITR gene clusters at the sequence level only have been performed with the zebrafish and medaka genomes [8–11]. Although transcripts of various catfish NITRs have been detected in NK-like, T, B, and macrophage cell lines [12], transcripts of all zebrafish NITRs are detectable in the lymphoid, but not the myeloid, lineage [13]. Of the 39 NITR genes that have been identified within the zebrafish genome, *nitr9* is the only NITR gene that is predicted to encode an activating receptor [10, 11, 14]. Three alternatively spliced transcripts of *nitr9* have been characterized: Nitr9-long (Nitr9L), Nitr9-short (Nitr9S), and Nitr9-supershort (Nitr9SS), which differ in their extracellular domains [13, 14]. Nitr9L is the most similar to other NITRs in that it possesses two extracellular Ig domains: one of the variable (V) type and one of the intermediate (I) type [6]. Nitr9S arises through cryptic splice donor and acceptor sites within the exon encoding the V domain. Nitr9SS lacks the entire V domain exon. The transmembrane domain of all Nitr9 isoforms possesses a positively charged residue: this feature permits Nitr9L to associate with and signal through the adaptor protein Dap12 [14]. Based on protein structures, Nitr9S and Nitr9SS also are expected to signal via Dap12; however, this has not been verified experimentally.

Although *nitr9* transcripts have been detected in zebrafish lymphocytes, the identification and recovery of Nitr9-expressing cells has not been possible. Herein we describe the derivation of two anti-Nitr9 monoclonal antibodies, demonstrate their utility to recognize recombinant Nitr9 by indirect immunofluorescence, flow cytometry, and Western blot analyses, and subsequently identify all three Nitr9 isoforms in zebrafish tissues by Western blot analyses. These antibodies should prove useful for: (1) evaluating Nitr9 protein levels within tissues by Western blot, (2) evaluating the distribution of Nitr9 expressing cells within tissues by indirect immunofluorescence, (3) defining the specific hematopoietic lineage(s) that express Nitr9 by flow cytometry, and (4) purifying Nitr9 expressing cells by fluorescence-activated cell sorting (FACS) for functional characterization.

## 2. Materials and Methods

### 2.1. Zebrafish

All experiments involving live zebrafish (*Danio rerio*) were performed in accordance with relevant institutional and national guidelines and regulations and were approved by the North Carolina State University Institutional Animal Care and Use Committee. Adult zebrafish (EkkWill Waterlife Resources, Ruskin, FL) were maintained and sacrificed as described [15].

### 2.2. Reverse Transcriptase-PCR

Total RNA from dissected zebrafish tissues (2 *μ*g) was reverse transcribed (SuperScript III Reverse Transcriptase, Life Technologies, Carlsbad, CA), and cDNAs were subjected to thermal cycling with gene-specific primers (Table 1) and Titanium *Taq* DNA polymerase (Clontech, Mountain View, CA). The number of PCR cycles used for detecting nitr9 and *β*-actin (both annealing at 65°C) was 40 and 25, respectively.

Lymphoid and myeloid cell populations were purified from the kidney of multiple zebrafish and pooled as described [16]. Total RNA from isolated cells (1 *μ*g) was reverse transcribed (SuperScript III Reverse Transcriptase). cDNAs from tissues and isolated cells were subjected to quantitative PCR (Q-PCR) with TaqMan primers and probes (Life Technologies, Carlsbad, CA) (Table 1). Q-PCR was performed on a single-color MyiQ real-time PCR detection system (Bio-Rad, Hercules, CA) using the protocol: 50°C for 2 min, 95°C for 10 min, followed by 55 cycles at 95°C for 15 s and at 60°C for 1 min. The threshold cycle (C_T_) value was calculated by the iQ5 Optical System Software (Bio-Rad). Relative transcript levels of nitr9 were normalized to *β*-actin and calculated using the 2^−ΔΔC_T_^ method [17]. All reactions were carried out as technical triplicates.

### 2.3. Antibody Development and Purification

The coding sequence of the Nitr9 I domain (nucleotides 298–623 of GenBank NM_001005576.1) was amplified by PCR (Table 1) and cloned into pETBlue-1 (EMD Millipore, Billerica, MA), and *E. coli* Tuner cells (EMD Millipore) were transformed employing a standard procedure. Cells were induced, and the Nitr9 I domain was recovered from inclusion bodies.

Swiss Webster mice were immunized with the Nitr9 I domain expressed in *E. coli* and splenocytes were fused with P3X63Ag8.653 cells (CRL-1580, ATCC, Manassas, VA). Approximately 3,000 individual hybridoma supernatants were screened by an enzyme-linked immunosorbent assay (ELISA) against the denatured recombinant Nitr9 I domain (Immunology Core Facility, University of North Carolina, Chapel Hill). The most strongly reactive ~100 supernatants in turn were screened by parallel Western blot analyses and indirect immunofluorescence. Two single clones, 19.1.1 (herein referred to as anti-Nitr9^19^) and 90.10.5 (herein referred to as anti-Nitr9^90^), were selected for additional characterization based on their ability to recognize recombinant Nitr9. Antibody isotypes were determined (IsoStrips: Roche; Indianapolis, IN) to be IgG2b, *κ* light chain (90.10.5), and IgG2a, *κ* light chain (19.1.1). Antibodies were purified via protein A agarose columns (Upstate Cell Signaling Solutions; Lake Placid, NY).

### 2.4. Plasmids and Cell Culture

**Figure 1:**
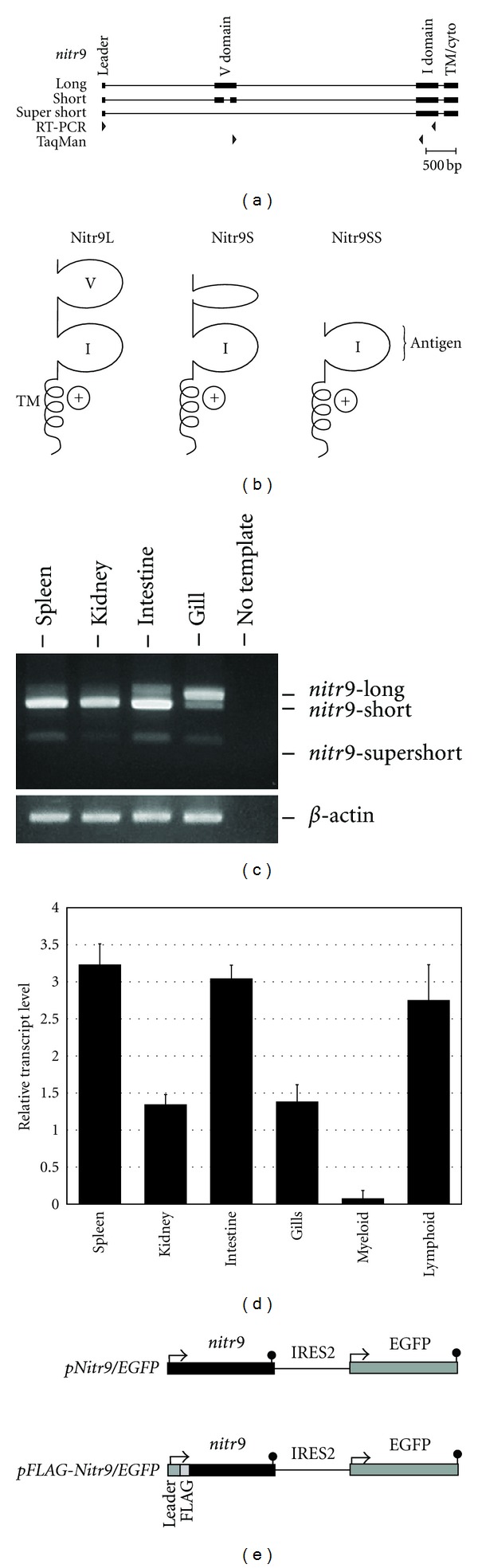
Transcriptional variation and expression of Nitr9. (a) Exon organization of the *nitr9* gene depicting the three transcript variants. Primer positions for PCR are indicated below. (b) The predicted Nitr9 protein isoforms encoded by the three *nitr9* transcripts. Transmembrane (TM) and immunoglobulin domains (of the variable (V) and intermediate (I) types) of Nitr9 are indicated. The I domain of Nitr9 was used as the antigen for antibody production. The positive charge within the TM domain of Nitr9 is represented by a plus sign. (c) RT-PCR with primers whose positions are depicted in (a) detects transcripts of all three *nitr9* isoforms. (d) Quantitative RT-PCR with *nitr9* primers (Table 1), whose positions are depicted in (a), and a TaqMan probe that spans an exon-exon boundary reveal relative levels of *nitr9L/S* transcripts in different tissues. (e) Schematic representation of the recombinant Nitr9 expression constructs used in this paper. Constructs include an internal ribosomal entry sequence (IRES2) permitting the expression of two proteins from a single transcript.

Nitr9 expression cassettes (without epitope tags) were constructed with *pcDNA3 *(Life Technologies). Epitope (FLAG)-tagged Nitr9 (FLAG-Nitr9) expression cassettes were constructed with the *pLF* plasmid which incorporates an amino-terminal leader sequence and FLAG epitope [14]. The coding sequences of zebrafish *nitr9L, nitr9S, *and* nitr9SS* were amplified by PCR and cloned into *pcDNA3 *or* pLF.* Nitr9 and FLAG-Nitr9 cassettes were then shuttled into *pIRES2-EGFP *(Clontech) generating: *pNitr9L/EGFP*, *pNitr9S/EGFP, pNitr9SS/EGFP, pFLAG-Nitr9L/EGFP*, *pFLAG-Nitr9S/EGFP, *and *pFLAG-Nitr9SS/EGFP* plasmids (Figure 1). Primer sequences that were used in cloning steps are included in Table 1. Plasmids were transfected into human HEK293T cells using Fugene 6 (Roche) according to the manufacturer's instructions and were harvested 48 hr after transfection. 

### 2.5. Indirect Immunofluorescence

HEK293T cells were transfected in four well chamber slides (Thermo Fisher Scientific, Rochester, NY). Transfected cells were washed in phosphate buffered saline (PBS), fixed with 3% paraformaldehyde for 20 min and treated with 50 mM NH_4_Cl, PBS for 5 minutes. Cells were then permeabilized with 1.0% Triton-X-100 in PBS for 5 min, rinsed and blocked with 1% BSA in PBS for 5 min. Permeabilized cells were incubated with the anti-Nitr9^19^, anti-Nitr9^90^, or anti-FLAG antibody for 1 hr, rinsed with PBS, incubated with a phycoerythrin (PE) anti-mouse IgG antibody and DAPI (1 : 1000) for 1 hr, and washed with PBS. Chambers were removed from the slides, and coverslips were mounted using immunomount (Thermo Shandon, Pittsburgh, PA). Cells were photographed at 40x magnification using a Leica DM5000 microscope.

### 2.6. Flow Cytometry

Transfected HEK293T cells were incubated with the anti-Nitr9^19^, anti-Nitr9^90^, or anti-FLAG monoclonal antibody for 1 hr, washed in PBS, and incubated for 30 min with an allophycocyanin- (APC-) conjugated anti-mouse IgG secondary antibody. Labeled cells were washed and then fixed with 3% paraformaldehyde and subjected to flow cytometric analysis (BD FACSCalibur, BD Biosciences, San Jose, CA). 

### 2.7. Western Analyses

Transfected HEK293T cells were washed with PBS and lysed with mammalian protein extraction reagent (M-PER, Pierce, Rockford, IL). Kidney, spleen, intestine, and gills were removed from sacrificed adult zebrafish and collected directly into tissue protein extraction reagent (T-PER, Pierce) supplemented with protease inhibitors (Pierce) and homogenized. Lysates were centrifuged to remove nuclei, and cell debris and protein concentrations were determined (BCA Protein Assay, Pierce). Proteins were resolved on 12% SDS-polyacrylamide gels and transferred to polyvinylidene difluoride (PVDF) membranes for Western analyses. Membranes were washed in Tris-buffered saline with 0.1% Tween 20 (TBST) and incubated in blocking buffer (100 mM boric acid, 25 mM Na-Borate, 75 mM NaCl, 5% goat serum, and 5% dry milk powder) for 1 hr. Membranes were incubated overnight with primary antibodies in blocking buffer at 4°C. Primary antibodies include anti-Nitr9^90^, anti-FLAG (M2) mouse monoclonal antibody (Sigma-Aldrich, St. Louis, MO), anti-GFP mouse monoclonal antibody (Roche), and anti-GAPDH rabbit polyclonal antibody (AnaSpec, Fremont, CA). Membranes were washed in TBST, followed by incubation with blocking buffer and either horseradish peroxidase-conjugated anti-mouse IgG secondary antibody (Roche) or horseradish peroxidase-conjugated anti-rabbit IgG secondary antibody (Santa Cruz Biotechnology, Santa Cruz, CA). After washing with TBST, the Lumi-Light^PLUS^ western blotting substrate and detection system (Roche) was used to visualize reactivity.

### 2.8. Endoglycosidase Treatment

Cleared lysates (20 *μ*g) from transfected cells were incubated with N-Glycosidase F (PNGase F, New England Biolabs, Ipswich, MA) for 1 hr at 37°C. Cleared lysates (25 *μ*g) from zebrafish tissues were precipitated with OrgoSOL buffer (G-Biosciences, Maryland Heights, MO) and resuspended in PNGase buffer for treatment with PNGase F.

## 3. Results and Discussion

### 3.1. Nitr9 Isoforms

The genomic organization and predicted protein structures of Nitr9L, Nitr9S, and Nitr9SS are shown in Figures 1(a) and 1(b). All three isoforms are predicted to encode type I transmembrane cell surface receptors that possess a positively charged residue within the transmembrane domain. The *nitr9S* isoform is expressed at higher levels in the zebrafish spleen, kidney, and intestine than the *nitr9L* and *nitr9SS* isoforms, whereas, *nitr9L *transcripts are the most abundant isoform expressed in gills. Transcripts of *nitr9SS* are detected in all four tissues at reduced levels relative to the other isoforms (Figure 1(c)). Q-PCR (Table 1) was employed to determine the combined relative levels of *nitr9L* and *nitr9S* transcripts in these same tissues as well as in purified lymphoid and myeloid cells (the TaqMan primer/probe set employed in this paper does not detect *nitr9SS *transcripts). The combined relative expression level of *nitr9L *and* nitr9S* transcripts is consistently higher in intestine than in kidney and gill (Figure 1(d)). However, the relative expression level of *nitr9L *and* nitr9S* in spleen varied between biological replicates, ranging from levels matching those in intestine to lower levels as observed in kidney and gill. As reported previously, *nitr9* transcripts are present at much higher levels in zebrafish lymphocytes as compared to myeloid cells [13]. In order to generate monoclonal antibodies that could detect all three Nitr9 isoforms, mice were immunized with a bacterially expressed Nitr9 I domain (see Figure 1(b)), and hybridomas were screened for the production of antibodies that recognize recombinant Nitr9 by ELISA, Western blot and indirect immunofluorescence. Two clones, 19.1.1 (herein referred to as anti-Nitr9^19^) and 90.10.5 (herein referred to as anti-Nitr9^90^), were selected for further evaluation.

### 3.2. Detection of Nitr9 Isoforms in Transfected Cells by Indirect Immunofluorescence

**Figure 2:**
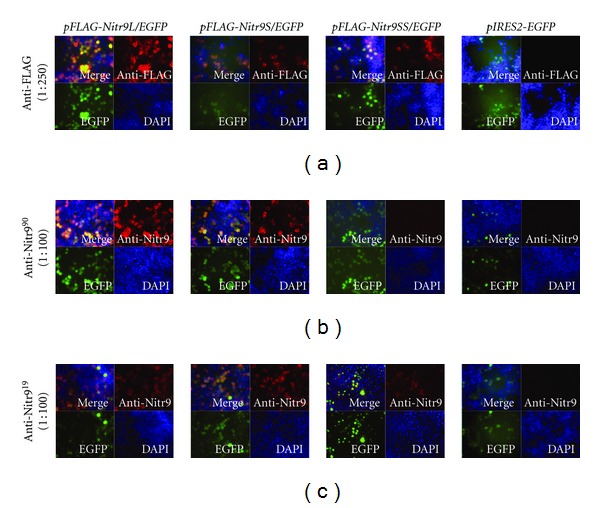
Detection of FLAG-tagged isoforms of Nitr9 from transfected cells by indirect immunofluorescence. HEK293T cells were transfected with plasmids encoding FLAG-tagged Nitr9 isoforms and EGFP as indicated on top of the panels. FLAG-tagged Nitr9 proteins were detected with (a) an anti-FLAG antibody, (b) anti-Nitr9^90^ or (c) anti-Nitr9^19^, and a PE conjugated secondary antibody (red). Transfected cells can be identified by EGFP expression (green). DAPI labels the nuclei of all cells (blue). The pIRES2-EGFP parental plasmid was included as a negative control.

**Figure 3:**
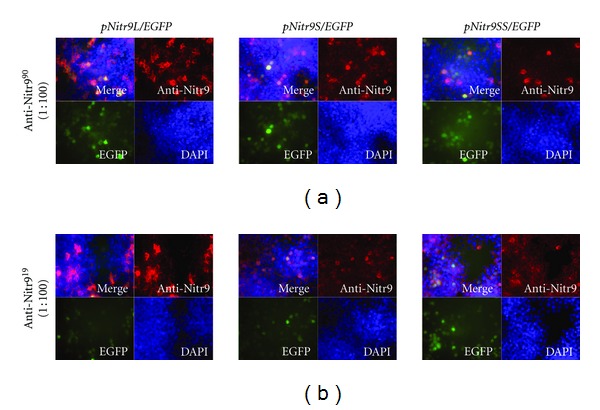
Detection of endogenous isoforms of Nitr9 from transfected cells by indirect immunofluorescence. HEK293T cells were transfected with plasmids encoding endogenous isoforms of Nitr9 and EGFP as indicated on top of the panels. Nitr9 proteins were detected with (a) anti-Nitr9^90^ or (b) anti-Nitr9^19^ and a PE conjugated secondary antibody (red). Transfected cells can be identified by EGFP expression (green). DAPI labels the nuclei of all cells (blue).

In order to determine if anti-Nitr9^19^ and anti-Nitr9^90^ could detect all three isoforms of Nitr9 by indirect immunofluorescence, HEK293T cells were transfected with plasmids that coexpress EGFP and either a FLAG-tagged or endogenous isoform of Nitr9; in this way, any cell expressing Nitr9 also expresses EGFP (Figure 1(e)). To ensure that the recombinant Nitr9 proteins could be detected by immunofluorescence, an anti-FLAG antibody was used to detect all three FLAG-tagged isoforms of Nitr9 in transfected cells (Figure 2(a)). It was then shown that both anti-Nitr9^19^ and anti-Nitr9^90^ recognize FLAG-Nitr9L and FLAG-Nitr9S by immunofluorescence, but either fail to bind (anti-Nitr9^90^) or bind less effectively (anti-Nitr9^19^) to FLAG-Nitr9SS (Figures 2(b) and 2(c)). In contrast, both anti-Nitr9^19^ and anti-Nitr9^90^ effectively recognize all three isoforms of endogenous Nitr9 when expressed in transfected cells albeit with an apparent higher background labeling of cells with anti-Nitr9^19^ (Figure 3). It is possible that the FLAG-tag disrupts folding of Nitr9SS or sterically interferes with antibody recognition of the I domain of FLAG-Nitr9SS; this also was observed with Western analyses (discussed below).

### 3.3. Detection of Nitr9 Isoforms in Transfected Cells by Flow Cytometry

**Figure 4:**
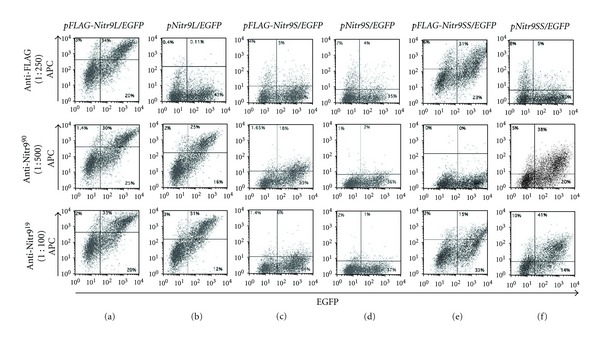
Detection of Nitr9 isoforms by flow cytometry. HEK293T cells were transfected with plasmids encoding FLAG-tagged (a, c, and e) or endogenous (b, d, and f) isoforms of Nitr9 and EGFP as indicated above the panels. Cells were labeled with an anti-FLAG antibody (top row), anti-Nitr9^90^ (middle row) or anti-Nitr9^19^ (bottom row), and an APC conjugated secondary antibody. Flow cytometric analyses were employed to detect EGFP positive (*X* axis) and APC positive (*Y* axis) cells. Isotype-matched antibodies were evaluated as controls for both anti-Nitr9 antibodies and displayed no labeling of transfected cells (data not shown).

In order to determine if anti-Nitr9^19^ and anti-Nitr9^90^ could detect all three isoforms of Nitr9 by flow cytometry, HEK293T cells were transfected with plasmids encoding an endogenous or FLAG-tagged isoform of Nitr9 and EGFP (Figure 1(e)). Flow cytometry was performed using the anti-FLAG, anti-Nitr9^19^, and anti-Nitr9^90^ antibodies to detect Nitr9 expressing cells. The percentage of double positive FLAG-Nitr9L expressing cells (i.e., EGFP^+^ and Nitr9^+^) was similar (55–63% of EGFP^+^ cells) when the anti-FLAG or the anti-Nitr9 antibodies were employed (Figure 4(a)). Both anti-Nitr9 antibodies recognize transfected cells expressing the endogenous isoform of Nitr9L with a similar efficiency (61–73% of EGFP^+^ cells) (Figure 4(b)).

The anti-FLAG monoclonal antibody failed to bind FLAG-Nitr9S and the anti-Nitr9^19^ antibody failed to detect the Nitr9S- or FLAG-Nitr9S-expressing cells (2–9% of EGFP^+^ cells) (Figures 4(c) and 4(d)). Although the anti-Nitr9^90^ antibody detects FLAG-Nitr9S (31% of EGFP^+^ cells), it does not recognize endogenous Nitr9S (~5% of EGFP^+^ cells). Although the Nitr9S and FLAG-Nitr9S proteins are produced by transfected cells (see Figures 2 and 3 and Western blot results below) they may not be expressed effectively on the cell surface. To determine if cell surface expression of Nitr9S requires coexpression of the signaling adaptor protein Dap12, cells were cotransfected with plasmids encoding Nitr9S and zebrafish Dap12. No increase was observed in cell surface labeling by the anti-Nitr9 antibodies (data not shown).

The anti-FLAG and anti-Nitr9^19^ antibodies effectively bound FLAG-Nitr9SS (57% and 31% of EGFP^+^ cells, resp.). The anti-Nitr9^90^ antibody failed to bind FLAG-Nitr9SS, possibly due to steric hindrance by the FLAG tag (Figure 4(e)) since both anti-Nitr9 antibodies were effective at recognizing Nitr9SS (65%–75% of EGFP^+^ cells; Figure 4(f)).

### 3.4. Anti-Nitr9^90^ Binds All Three Isoforms of Nitr9 in Western Analyses

**Figure 5:**
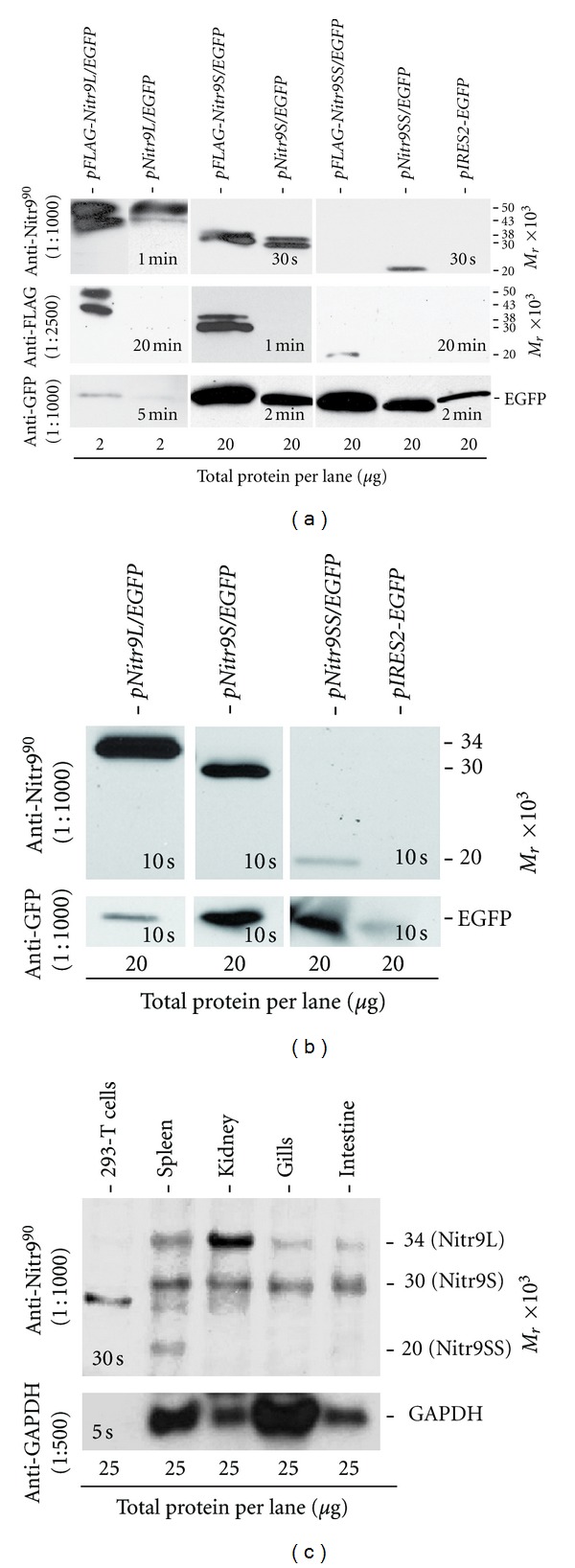
Detection of Nitr9 protein by Western analyses. (a) Western blot analyses of total protein lysates from HEK293T cells transiently transfected with plasmids expressing a Nitr9 isoform and EGFP. Plasmids encode either an endogenous isoform of Nitr9 or a FLAG-tagged Nitr9 as indicated above each lane. The primary antibodies utilized are shown on the left, and the molecular weights of identified bands are shown on the right. The anti-FLAG antibody serves as a positive control for Nitr9 detection, and the anti-GFP antibody indicates transfection efficiency of each plasmid. Note the total protein loaded (bottom) for the Nitr9L isoform is ten times less than that for Nitr9S and Nitr9SS plasmids. Exposure times for chemiluminescence detection are indicated in each panel. (b) Nitr9L and Nitr9S are glycosylated. Western blot analyses of endoglycosidase-treated total protein lysates from HEK293T cells that were transfected with plasmids encoding endogenous Nitr9 isoforms. The anti-Nitr9^90^ antibody recognizes all three Nitr9 isoforms at the predicted size (right). (c) Detection of Nitr9 protein from zebrafish tissues. Western blot analyses of 25 *μ*g of endoglycosidase-treated total protein from zebrafish tissues and HEK293T cells. Note that a nonspecific band (~28 kD) is detected in HEK293T cells as well as in zebrafish kidney and spleen, with high protein loads. Bottom panel indicates loading control using an anti-GAPDH polyclonal antibody.

In order to evaluate the ability of the anti-Nitr9^90^ antibody to detect the three isoforms of Nitr9 in Western analyses, HEK293T cells were transfected with plasmids encoding endogenous and FLAG-tagged isoforms of Nitr9 (Figure 1(e)). Cell lysates were subjected to Western blot analyses using the anti-Nitr9^90^ antibody. All three isoforms of the endogenous Nitr9 as well as the FLAG-tagged Nitr9L and Nitr9S proteins were detected. A binding pattern equivalent to that seen with the anti-FLAG monoclonal antibody positive control is apparent (Figure 5(a)). However, anti-Nitr9^90^ failed to bind the FLAG-tagged Nitr9SS. As mentioned above, this may be a result of the FLAG-tag blocking access to the specific epitope recognized by this antibody. 

Two proteins bands were detected by anti-Nitr9^90^ in both endogenous and FLAG-tagged Nitr9L and Nitr9S transfections that were also bound by the anti-FLAG antibody. Both observed Nitr9L proteins migrated at a higher molecular weight than the predicted size of Nitr9L (34 kD), and one of the observed Nitr9S proteins was larger than the predicted size of Nitr9S (30 kD). The differences are consistent with differential glycosylation (see below). Based on the chemiluminescence exposure times required for detecting the different isoforms of Nitr9, anti-Nitr9^90^ appears to exhibit a higher affinity for Nitr9L as compared to Nitr9S and Nitr9SS. In parallel experiments, the anti-Nitr9^19^ antibody did not bind endogenous Nitr9S and Nitr9SS proteins (data not shown) and was not characterized further in the Western blot analyses. 

### 3.5. Nitr9 Glycosylation in Transfected Cells

Nitr9L, Nitr9S and Nitr9SS possess three (NMSC, NDSR, and NGSK), two (NMSC and NGSK), and one (NGSK) candidate N-linked glycosylation sites, respectively. Treatment of lysates from Nitr9 transfected cells with endoglycosidase (PNGase F) results in the detection of only a single protein of the expected size for both Nitr9L and Nitr9S (Figure 5(b)). Both sets of results are consistent with *in vivo* glycosylation. The observed size of Nitr9SS in transfected cells does not appear to be altered by endoglycosidase treatment, with the limitations of detection, suggesting that it may not be glycosylated.

### 3.6. Nitr9 Proteins Are Differentially Expressed in Different Tissues of Zebrafish

In order to determine if the anti-Nitr9^90^ antibody can recognize endogenous Nitr9, lysates from adult zebrafish tissues were treated with endoglycosidase and subjected to Western blot analyses (Figure 5(c)). Nitr9L and Nitr9S were detected at varying levels in the spleen, kidney, gills, and intestine. Nitr9SS was detected only in the spleen, although faint bands also have been observed in intestine (data not shown). A nonspecific band of approximately 28 kD is detected in zebrafish tissues as well as in HEK293T cells when the anti-Nitr9^90^ antibody is used with large total protein loads (e.g., 25 *μ*g lysate; Figure 5(c)). 

## 4. Conclusions

Three different transcript variants from *nitr9, *the single putative activating NITR gene in zebrafish, and their corresponding protein isoforms have been identified and characterized. The utility of the anti-Nitr9^19^ and anti-Nitr9^90^ monoclonal antibodies for detecting recombinant Nitr9 was demonstrated by indirect immunofluorescence, flow cytometry, and Western blot analyses. The antibodies exhibit profound differences in recognizing the three different Nitr9 isoforms. When employed for indirect immunofluorescence, both anti-Nitr9 antibodies bound efficiently and specifically to cells-expressing all three Nitr9 isoforms. Both anti-Nitr9 antibodies are effective for detecting cell surface expression of Nitr9L and Nitr9SS by flow cytometry. The anti-Nitr9^90^ antibody recognized all three Nitr9 isoforms by Western blot analyses, although a higher affinity for Nitr9L is noted. When using anti-Nitr9^90^ in Western blot analyses with high levels of protein, a nonspecific band was identified. Although the identity of this protein remains unknown, it may represent a well-conserved member of the Ig superfamily. 

Marked differences in the relative levels of Nitr9 transcripts and protein isoforms are apparent. Although the PCR analyses (Figure 1(c)) suggest that *nitr9S* may be the predominant mRNA isoform in spleen, kidney, and intestine, Western analyses demonstrate that Nitr9L is the predominant protein isoform expressed in kidney. This discrepancy may reflect differing transcript and protein stability in different tissues or the preferred reactivity of the antibody with Nitr9L (Figure 5(a)).

The monoclonal antibodies described here should be useful for further evaluation of Nitr9 protein levels in zebrafish tissues by Western blot analyses and identifying Nitr9 expressing cells in tissue sections by indirect immunofluorescence. Efforts are underway to purify Nitr9-expressing zebrafish cells employing FACS in order to characterize their morphology and cytotoxic properties. These antibodies may also prove to be useful for activating (crosslinking) or blocking Nitr9 function in both cell culture and *ex-vivo* functional assays as well as in dissecting isoform-specific functions of NITRs. 

## Figures and Tables

**Table 1:** Oligonucleotide primer sequences.

Purpose	Primer sequence
Reverse transcriptase—PCR: *nitr9 *	GGATTTTTGGACTTTTCTGTC
	TCCACATGCGGTAACTGTAC
Reverse transcriptase—PCR: *β*-*actin *	GGTATGGAATCTTGCGGTATCCAC
	ATGGGCCAGACTCATCGTACTCCT
TaqMan Q-PCR: *nitr9* (probe = CAAGGTTTGGAAAAGCAC)	GTCAAAGGGACAAGGCTGATAGTT
	GTTCAAAACAGTGCATGTAAGACTCA
TaqMan Q-PCR: *β*-*actin* (probe = CCCATGCCATCCTGC)	CCATCTATGAGGGTTACGCTCTTC
	AGGATCTTCATCAGGTAGTCTGTCA
Amplify *nitr9* I domain for bacterial expression construct	A TGGAAAAGCACACTGTAGTA^a^
	**TTA**TTTAGAGCCATTCCTGTCC^b^
Amplify *nitr9L* for FLAG-tagged expression cassette	CACCCAAATGCACCACCTGTGTTTGTTAAAC^c^
	gactgcggccgcTTACTGCTGGTTAGAAAC^d^
Amplify *nitr9S* for FLAG-tagged expression cassette	CACCCAAATGCACCACCTGTG^c^
	gactgcggccgcTTACTGCTGGTTAGAAAC^d^
Amplify *nitr9SS* for FLAG-tagged expression cassette	CATGATTTAATTCCATCCCA^c^
	gactgcggccgcTTACTGCTGGTTAGAAAC^d^
Amplify wild type *nitr9L*, *nitr9S* and *nitr9SS* for expression cassettes	gatcggatccgacATGATCAACTTTTGGATTT^e^
	gatcgaattcTTACTGCTGGTTAGAAACCGAG^f^

^a^An artificial start codon is underlined.

^b^An artificial stop codon is bold.

^c^These primers are designed for blunt PCR cloning into the *Eco*RV site of pLF.

^d^Overhang (5′) sequences are in lower case text and include a *Not* I site for cloning into pLF.

^e^Overhang (5′) sequences are in lower case text and include a *Bam*HI site for cloning into pcDNA3.

^f^Overhang (3′) sequences are in lower case text and include an *Eco*RI site for cloning into pcDNA3.
